# Prognostic implications of regression of metastatic axillary lymph nodes after neoadjuvant chemotherapy in patients with breast cancer

**DOI:** 10.1038/s41598-021-91643-z

**Published:** 2021-06-09

**Authors:** Yul Ri Chung, Ji Won Woo, Soomin Ahn, Eunyoung Kang, Eun-Kyu Kim, Mijung Jang, Sun Mi Kim, Se Hyun Kim, Jee Hyun Kim, So Yeon Park

**Affiliations:** 1 Department of Pathology, Seoul National University Bundang Hospital, Seoul National University College of Medicine, 82, Gumi-ro 173 Beon-gil, Bundang-gu, Seongnam, 13620 Gyeonggi Republic of Korea; 2 Pathology Center, Seegene Medical Foundation, Seoul, Republic of Korea; 3 Department of Surgery, Seoul National University Bundang Hospital, Seoul National University College of Medicine, Seongnam, Gyeonggi Republic of Korea; 4 Department of Radiology, Seoul National University Bundang Hospital, Seoul National University College of Medicine, Seongnam, Gyeonggi Republic of Korea; 5 Division of Hematology and Medical Oncology, Department of Internal Medicine, Seoul National University Bundang Hospital, Seoul National University College of Medicine, Seongnam, Gyeonggi Republic of Korea

**Keywords:** Surgical oncology, Breast cancer, Prognostic markers

## Abstract

Prognostic implications of therapeutic response of metastatic lymph nodes (LNs) to neoadjuvant chemotherapy (NAC) remain unclear in patients with breast cancer. We aimed to evaluate the prognostic value of axillary LN regression after NAC in locally-advanced breast cancer patients. Therapeutic response of the LNs was evaluated in 563 breast cancer patients and classified into four grades according to the regression pattern. Initial pathologic N stage was estimated from the sum of the metastatic LNs and those with complete regression. In survival analyses, LN regression grade, pathologic N stage after NAC, and presumed initial pathologic N stage stratified clinical outcome of the patients in the whole group, in both ER-positive and ER-negative subgroups, and in those with residual breast disease. On multivariate analysis, LN regression grade and presumed initial pathologic N stage were revealed as independent prognostic factors. The number of completely-responsive LNs and the ratio of non-responsive LNs also revealed a prognostic value. In conclusion, regression grade of axillary LNs and presumed initial pathologic N stage have prognostic values in breast cancer patients who receive NAC. Thus, regression of axillary LNs should be evaluated and included in pathologic reporting of post-NAC resection specimens.

## Introduction

Neoadjuvant chemotherapy (NAC) is regarded a standard therapy in patients with locally advanced breast cancer. Advantages of NAC include downstaging which allows inoperable tumors surgically-removable, reducing the extent of surgery, preventing systemic metastasis, and obtaining information on chemo-responsiveness^1^.

Pathologic complete response (pCR), defined as no residual invasive disease in both the breast and axilla after NAC, is a well-established prognostic factor in patients with breast cancer^2^. However, a significant proportion of patients who receive NAC does not reach pCR, and those who do not reach pCR include a broad range of patients with different responses to NAC. Thus, more detailed indices including Miller-Payne grading system which reflect cellularity difference between pre- and post-treatment tumors and the Residual Cancer Burden (RCB) classification that incorporates both primary and axillary tumor burden have been devised^3,4^. These systems not only reflect chemo-responsiveness of a tumor but also predict patients’ clinical outcome^4,5^.

Axillary status after NAC has been consistently reported as a robust prognostic factor for patient survival independent of primary tumor response^6,7^. In addition to the absolute number of residual metastatic LNs, some have reported the prognostic significance of the LN ratio (number of metastatic LNs divided by number of dissected LNs) in NAC setting^8–12^. However, the prognostic value of LN regression grade has not been studied widely in breast cancer.

In this study, we evaluated the regression of metastatic LNs, estimated initial pathologic N stage, and finally assessed their prognostic value in predicting survival of the patients with locally advanced breast cancer treated with NAC.

## Results

### Clinicopathologic characteristics

This study included either clinical stage II (*n* = 285) or III (*n* = 278) breast cancer patients treated with NAC. As for estrogen receptor (ER) status, 352 patients (62.5%) were ER-positive while the remaining 211 patients (37.5%) were ER-negative. For progesterone receptor (PR) status, 293 patients (52.0%) were PR-positive, and 270 (48.0%) were PR-negative. As for human epidermal growth receptor 2 (HER 2), 167 patients (29.7%) were HER2-positive while 396 patients (70.3%) were HER2-negative. The AC regimen consisting of doxorubicin and cyclophophamide for 4 to 6 cycles was given to 142 patients (25.2%), sequential AC-T comprising 4 cycles of AC followed by 4 cycles of docetaxel was administered in 271 patients (48.1%), and sequential AC-TH comprising 4 cycles of AC followed by 4 cycles of docetaxel and trastuzumab was given to 54 patients (9.6%). The AD regimen consisting of doxorubicin and docetaxel for 3 to 6 cycles was administered in 70 patients (12.4%), and the remaining 26 patients (4.6%) received various other regimens. The patients received breast surgery 3–4 weeks after the last chemotherapy cycle. Table 1 summarizes the clinicopathological features prior to NAC.

**Table 1:** Clinicopathological characteristics of study population.

Clinicopathological characteristics	No. (%)
**Age (years old)**	
< 50	331 (58.8)
≥ 50	232 (41.2)
**Clinical stage**	
II	285 (50.6)
III	278 (49.4)
**Clinical T stage**	
cT1	42 (7.5)
cT2	304 (54.0)
cT3	161 (28.6)
cT4	56 (9.9)
**Clinical N stage**	
cN0	95 (16.9)
cN1-N3	468 (83.1)
**Histologic subtype**	
IDC	524 (93.1)
ILC	18 (3.2)
Metaplastic carcinoma	7 (1.2)
Mucinous carcinoma	6 (1.1)
Others	8 (1.4)
**Histologic grade**	
Low to intermediate	330 (58.6)
High	233 (41.4)
**Estrogen receptor**	
Negative	211 (37.5)
Positive	352 (62.5)
**Progesterone receptor**	
Negative	270 (48.0)
Positive	293 (52.0)
**HER2 status**	
Negative	396 (70.3)
Positive	167 (29.7)
**Ki-67 proliferation index**	
< 20%	190 (33.7)
≥ 20%	373 (66.3)
**Molecular subtype**	
Luminal A	120 (21.3)
Luminal B	237 (42.1)
HER2 +	79 (14.0)
Triple-negative	127 (22.6)
**Chemotherapy regimen**	
AC	142 (25.2)
AD	70 (12.4)
AC-T	271 (48.1)
AC-TH	54 (9.6)
Others	26 (4.6)

*IDC* invasive ductal carcinoma, *ILC* invasive lobular carcinoma, *AC* doxorubicin plus cyclophosphamide, *AD* doxorubicin plus docetaxel, *AC-T* AC followed by docetaxel, *AC-TH* AC followed by docetaxel and trastuzumab.

### Chemotherapeutic response of LNs after NAC

**Figure 1:**
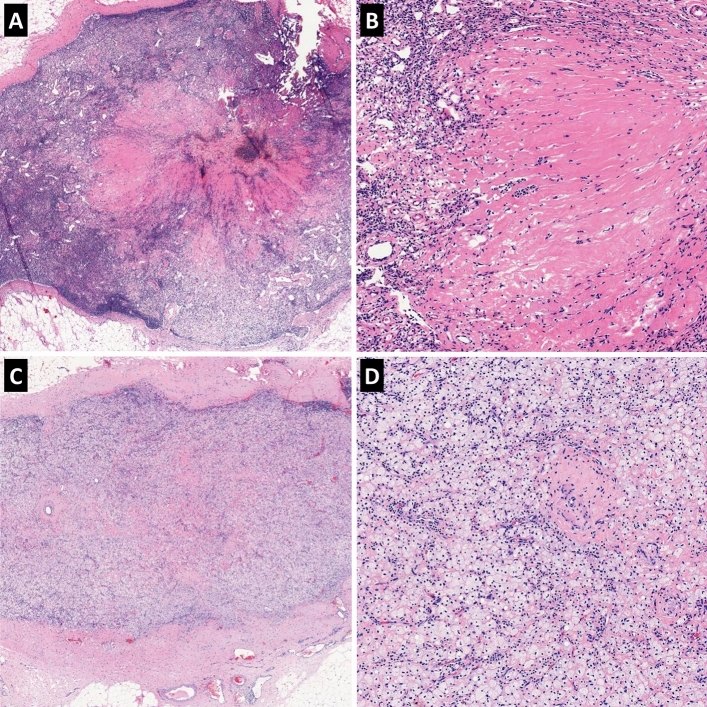
Representative images of complete tumor regression in axillary lymph nodes. **(A)** A large area of intranodal fibrosis is noted in the center of lymph node. **(B)** Magnification of A reveals depletion of lymphocytes and replacement with dense fibrous tissue. **(C)** Nodal architecture is obliterated with infiltration of foamy histiocytes and intranodal fibrosis. Thickened lymph node capsule is also found. **(D)** Magnification of **(C)** shows heavy infiltration of foamy histiocytes.

Prior to NAC, 95 (16.9%) of the 563 patients showed no evidence of nodal metastases (cN0) while 468 (83.1%) patients were suspected of having nodal metastases by imaging studies (cN1–N3). After NAC, axillary LN metastasis was found in 298 patients (52.9%), and the mean number of metastatic LNs was 2.87 (standard deviation, 5.35). The mean ratio of non-responsive lymph nodes to the total number of estimated metastatic LNs was 0.61 (standard deviation, 0.45). As for LN regression grade, 136 (24.2%) patients had no metastatic LNs without regression (grade 0), 129 (22.9%) patients had no metastatic LNs with complete regression (grade 1; Fig. 1), 78 (13.9%) patients had metastatic LNs but complete regression in some LNs (grade 2), and 220 (39.1%) patients had metastatic LNs with no regression (grade 3). Initial pathologic N (ipN) stage was estimated from the sum of the number of metastatic LNs and those with a complete regressive change. There were 136 (24.2%) cases with ipN0, 232 (41.2%) cases with ipN1, 136 (24.2%) cases with ipN2, and 59 (10.5%) cases with ipN3. Table 2 summarizes the clinicopathological characteristics of tumor after NAC including response of LNs.

**Table 2:** Clinicopathological characteristics of tumors after neoadjuvant chemotherapy.

Clinicopathological characteristics	No. (%)
**Pathologic T stage after NAC**	
ypT0	86 (15.3)
ypTis	40 (7.1)
ypT1	240 (42.6)
ypT2	161 (28.6)
ypT3	27 (4.8)
ypT4	9 (1.6)
**Pathologic N stage after NAC**	
ypN0	265 (47.1)
ypN1	159 (28.2)
ypN2	91 (16.2)
ypN3	48 (8.5)
**Miller-Payne regression grade**	
Grade 1	30 (5.3)
Grade 2	89 (15.8)
Grade 3	252 (44.8)
Grade 4	66 (11.7)
Grade 5	126 (22.4)
**Lymph node regression grade**	
Grade 0	136 (24.2)
Grade 1	129 (22.9)
Grade 2	78 (13.9)
Grade 3	220 (39.1)
**Estimated initial pathologic N stage**^**a**^	
ipN0	136 (24.2)
ipN1	232 (41.2)
ipN2	136 (24.2)
ipN3	59 (10.5)
**Residual cancer burden class**	
Class 0	113 (20.1)
Class I	45 (8.0)
Class II	174 (30.9)
Class III	231 (41.0)
**Lymphovascular invasion**^**b**^	
Absent	236 (54.0)
Present	201 (46.0)
**Estrogen receptor**^**b**^	
Negative	126 (28.8)
Positive	311 (71.2)
**Progesterone receptor**^**b**^	
Negative	221 (50.6)
Positive	216 (49.4)
**HER2 status**^**b**^	
Negative	317 (72.5)
Positive	120 (27.5)
**Ki-67 proliferation index**^**b**^	
< 20%	315 (72.1)
≥ 20%	122 (27.9)

*NAC* neoadjuvant chemotherapy.

^a^Initial pathologic N stage was estimated from the sum of number of positive lymph nodes and those with a regressive change.

^b^These characteristics were evaluated in cases with residual disease in breast.

### Clinicopathologic characteristics of tumors in relation to chemo-responsiveness of LNs

The clinicopathologic characteristics of the tumors according to chemo-responsiveness of the LNs are shown in Table 3. Briefly, grade 1 (complete regression) was significantly associated with high histologic grade, negative ER and PR status, and high Ki-67 index prior to NAC compared to grade 2 (partial regression) or grade 3 (no regression) (all *P* < 0.05). In addition, ypT0-T1, Miller-Payne grades 4–5, RCB classes 0-I, and absence of lymphovascular invasion after NAC were also associated with complete LN response to NAC (all *P* < 0.05). Conversely, in the LN non-responsive tumors encompassing grades 2 and 3, low to intermediate histologic grade, positive ER and PR status, low Ki-67 index, ypT2-T4, Miller-Payne grades 1–3, RCB classes II-III, and presence of lymphovascular invasion were more common as compared to completely node-responsive tumors (all *P* < 0.05). Grade 2 and grade 3 also showed difference in clinicopathologic characteristics with negative ER and PR status, positive HER2 status, high Ki-67 index, ypT0-T1, Miller-Payne grades 4–5, and RCB classes 0-I being more frequent in grade 2 than in grade 3 (all *P* < 0.05). As for breast cancer subtype, luminal A and luminal B subtypes were most prevalent in grade 3, and HER2 + and triple-negative subtypes were most frequent in grade 1.

**Table 3:** Clinicopathological characteristics of tumors according to chemo-responsiveness of lymph nodes.

Clinicopathological characteristics	Grade 1 (complete regression)	Grade 2 (partial regression)	Grade 3 (no regression)	*p* value			
				Among three grades	Grade 1 vs. grade 2^a^	Grade 2. vs. grade 3^a^	Grade 1 vs. grade 3^a^
***Before NAC***							
**Clinical T stage**				0.069	0.297	0.063	1.000
T1–T2	78 (60.5)	38 (48.7)	140 (63.6)				
T3–T4	51 (39.5)	40 (51.3)	80 (36.4)				
**Clinical N stage**				0.471	0.978	1.000	1.000
N0	8 (6.2)	2 (2.6)	13 (5.9)				
N1–N3	121 (93.8)	76 (97.4)	207 (94.1)				
**Histologic grade**				< 0.001	< 0.001	0.969	< 0.001
Low to intermediate	51 (39.5)	51 (65.4)	157 (71.4)				
High	78 (60.5)	27 (34.6)	63 (28.6)				
**Estrogen receptor**				< 0.001	0.024	0.003	< 0.001
Negative	74 (57.4)	30 (38.5)	44 (20.0)				
Positive	55 (42.6)	48 (61.5)	176 (80.0)				
**Progesterone receptor**				< 0.001	0.033	0.018	< 0.001
Negative	86 (66.7)	38 (48.7)	69 (31.4)				
Positive	43 (33.3)	40 (51.3)	151 (68.6)				
**HER2 status**				< 0.001	0.471	< 0.001	< 0.001
Negative	63 (48.8)	46 (59.0)	183 (83.2)				
Positive	66 (51.2)	32 (41.0)	37 (16.8)				
**Ki-67 index before NAC**				< 0.001	< 0.001	0.006	< 0.001
Low (< 20%)	14 (10.9)	24 (30.8)	112 (50.9)				
High (≥ 20%)	115 (89.1)	54 (69.2)	108 (49.1)				
**Breast cancer subtype**				< 0.001	0.003	< 0.001	< 0.001
Luminal A	5 (3.9)	12 (15.4)	78 (35.5)				
Luminal B	50 (38.8)	39 (50.0)	99 (45.0)				
HER2 +	33 (25.6)	17 (21.8)	16 (7.3)				
Triple-negative	41 (31.8)	10 (12.8)	27 (12.3)				
***After NAC***							
**ypT stage**				< 0.001	< 0.001	0.006	< 0.001
T0–T1	118 (91.5)	50 (64.1)	96 (43.6)				
T2–T4	11 (8.5)	28 (35.9)	124 (56.4)				
**Miller-Payne grade**				< 0.001	< 0.001	< 0.001	< 0.001
Grade 1–3	28 (21.7)	57 (73.1)	201 (91.4)				
Grade 4–5	101 (78.3)	21 (26.9)	19 (8.6)				
**RCB class**				< 0.001	< 0.001	0.045	< 0.001
Class 0–I	100 (77.5)	5 (6.4)	2 (0.9)				
Class II–III	29 (22.5)	73 (93.6)	218 (99.1)				
**Lymphovascular invasion**^**b**^				0.002	0.009	1.000	0.003
Absent	36 (69.2)	30 (42.3)	93 (43.5)				
Present	16 (30.8)	41 (57.7)	121 (56.5)				
**Ki-67 index after NAC**^**b**^				0.394	2.661	0.882	0.801
Low (< 20%)	36 (69.2)	50 (70.4)	164 (76.6)				
High (≥ 20%)	16 (30.6)	21 (29.6)	50 (23.4)				

*NAC* neoadjuvant chemotherapy, *RCB* residual cancer burden.

*P* values were calculated by the chi-square test or Fisher's exact test.

Numbers in parentheses indicate percentage.

^a^Corrections for multiple testing were made by Bonferroni method, and adjusted (adj.) p values were presented.

^b^These characteristics were evaluated in cases with residual disease in breast.

### Prognostic significance of chemo-responsiveness of LNs

**Figure 2:**
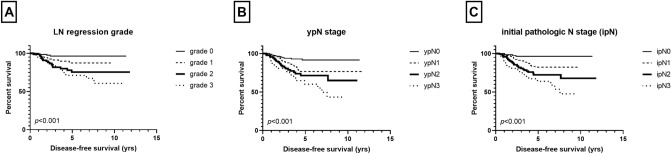
Disease-free survival based on lymph node (LN) status after neoadjuvant chemotherapy. There is a stepwise decline in survival with each increase in LN regression grade **(A)**, ypN stage **(B)**, and presumed initial pathologic N stage **(C)** in the whole group.

Survival analysis of the 563 patients revealed an association between four LN regression grades and disease-free survival of the patients, with grade 0 having the most favorable survival and grade 3 displaying the worst survival (*p* < 0.001, as a whole; *p* = 0.049, grade 0 vs. grade 1; *p* = 0.061, grade 1 vs. grade 2; *p* = 0.377, grade 2 vs. grade 3; Fig. 2). Pathologic N stage after NAC (ypN) also showed an association with disease-free survival (*p* < 0.001, as a whole; *p* = 0.003, ypN0 vs. ypN1; *p* = 0.157, ypN1 vs. ypN2; *p* = 0.146, ypN2 vs. ypN3; Fig. 2). As for ipN stage before NAC, we observed that ipN0 had the longest survival, and there was an almost stepwise decline in survival with each increase in ipN stage (*p* < 0.001, as a whole; *p* = 0.013, ipN0 vs. ipN1; *p* = 0.012, ypN1 vs. ypN2; *p* = 0.096, ypN2 vs. ypN3; Fig. 2).

**Figure 3:**
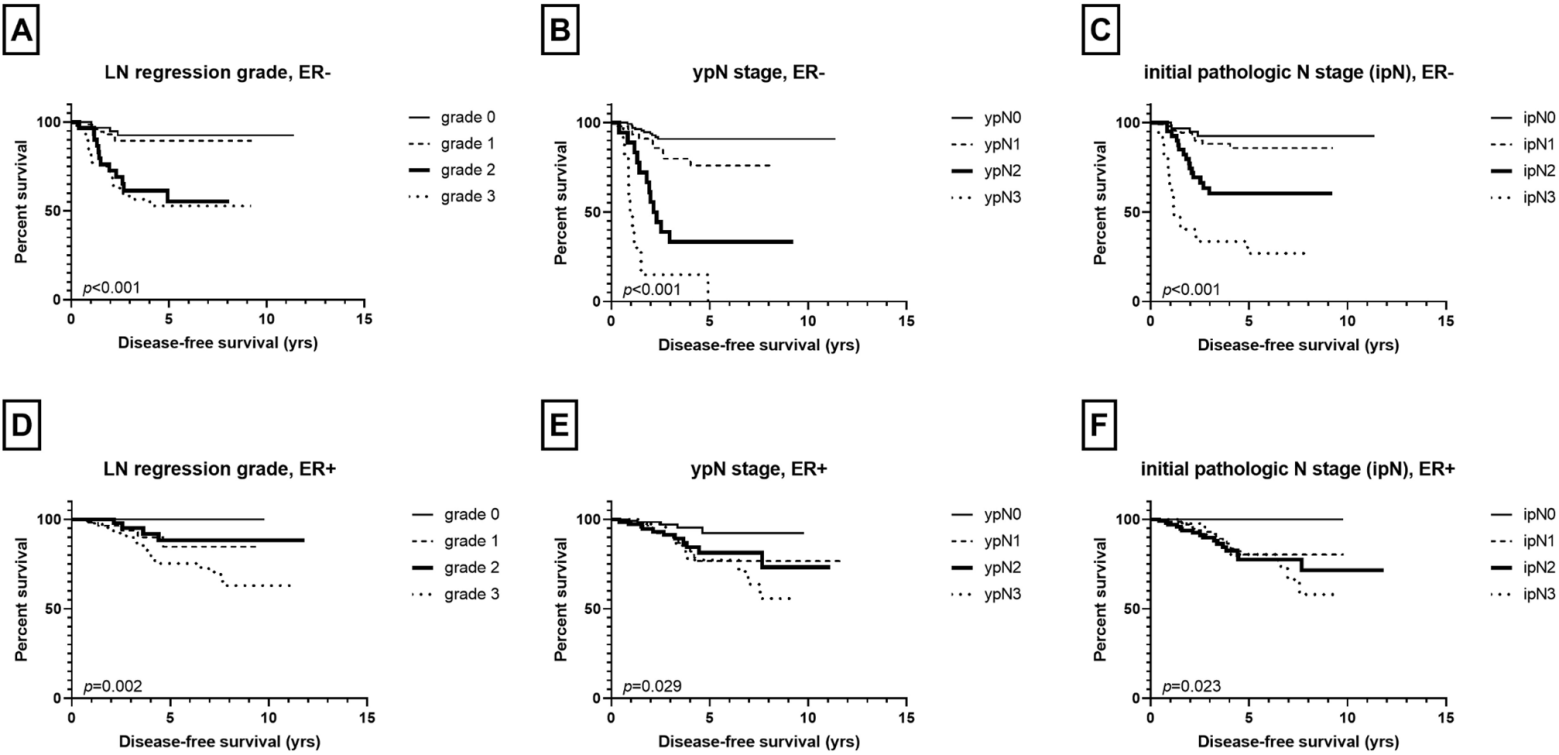
Disease-free survival based on lymph node (LN) status after neoadjuvant chemotherapy by estrogen receptor (ER) status. In ER-negative subgroup, significant survival difference is observed based on LN regression grade **(A)**, ypN stage **(B)**, and initial pathologic N (ipN) stage **(C)**, while in ER-positive subgroup, LN regression grade **(D)**, ypN stage **(E)**, and ipN stage **(F)** show less significant associations with patients’ survival.

Next, we performed the same set of analyses according to ER status. In ER-negative group (n = 211), there was a significant difference in disease-free survival among the four regression grades as a whole (*p* < 0.001; Fig. 3). However, grades 0 and 1 showed similar favorable survival while grades 2 and 3 revealed poor survival alike (*p* = 0.524, grade 0 vs. grade 1; *p* = 0.001, grade 1 vs. grade 2; *p* = 0.604, grade 2 vs. grade 3). As for pathologic N stage after NAC, there was a stepwise decrease in survival with increased ypN stage (*p* < 0.001, as a whole; *p* = 0.043, ypN0 vs. ypN1; *p* = 0.001, ypN1 vs. ypN2; *p* = 0.006, ypN2 vs. ypN3; Fig. 3). Presumed initial pathologic pN stage also showed an association with disease-free survival as a whole (*p* < 0.001; Fig. 3), but there was no survival difference between ipN0 and ipN1 (*p* = 0.361, ipN0 vs. ipN1; *p* = 0.001, ypN1 vs. ypN2; *p* = 0.003, ypN2 vs. ypN3).

In ER-positive group (n = 352), LN regression grade, ypN stage, and ipN stage were associated with disease-free survival of the patients as a whole (*p* = 0.002, *p* = 0.029, *p* = 0.023; Fig. 3), but there was no survival difference between LN regression grades 1 and 2, between ypN1, ypN2, and ypN3 or between ipN1, ipN2, and ipN3.

When we analyzed the association between LN status and survival in the patient group with residual breast disease, we also observed a difference in survival with respect to LN regression grade, ypN stage, and ipN stage (*p* = 0.005, *p* = 0.001, *p* < 0.001, respectively).

### Multivariate analyses including chemo-responsiveness of LNs

We performed multivariate analyses to evaluate whether nodal response to NAC had an independent prognostic value. In the whole group, ypT stage (*p* < 0.001), Miller-Payne regression grade (*p* < 0.001), and RCB class (*p* < 0.001) were associated with disease-free survival as well as LN regression grades, ypN stage, and ipN stage. As LN regression grades, ypN stage, and ipN stage were highly correlated with each other (LN regression grades and ypN stage, r = 0.775; LN regression grades and ipN stage, r = 0.663; ypN stage and ipN stage, r = 0.801), multivariate analyses were conducted in different modes. In multivariate analysis, LN regression grade and ipN stage, but not ypN stage were revealed as independent prognostic factors (*p* = 0.016, *p* = 0.006, *p* = 0.097, respectively; Table 4).

**Table 4:** Multivariate analyses of disease-free survival in the whole group.

Model	Variable	Category	Multivariate analysis		
			HR	95% CI	*p* value
A	ypT stage	T0–1 vs. T2–4	2.043	1.283–3.253	0.003
	Miller-Payne grade	Grade 4–5 vs. 1–3	0.875	0.354–2.160	0.772
	RCB class	0–I vs. II–III	6.952	2.143–22.551	0.001
	LN regression grade				0.016
	Grade 0		Reference		
	Grade 1		6.670	2.080–21.387	0.001
	Grade 2		3.745	1.247–11.248	0.019
	Grade 3		4.119	1.482–11.449	0.007
B	ypT stage	T0–1 vs. T2–4	2.122	1.340–3.360	0.001
	Miller-Payne grade	Grade 4–5 vs. 1–3	0.882	0.362–2.153	0.783
	RCB class	0–I vs. II–III	5.125	1.797–14.611	0.002
	ypN stage				0.097
	ypN0		Reference		
	ypN1		1.401	0.718–2.734	0.323
	ypN2		1.599	0.789–3.240	0.193
	ypN3		2.511	1.196–5.271	0.015
C	ypT stage	T0–1 vs. T2–4	1.711	1.082–2.897	0.023
	Miller-Payne grade	Grade 4–5 vs. 1–3	1.052	0.432–2.562	0.911
	RCB class	0-I vs. II–III	4.471	1.564–12.783	0.005
	ipN stage				0.006
	ipN0		Reference		
	ipN1		3.592	1.258–10.259	0.017
	ipN2		4.400	1.535–12.611	0.006
	ipN3		6.603	2.239–19.469	0.001

*HR* hazard ratio, *CI* confidence interval, *RCB* residual cancer burden, *ipN stage* initial pathologic N stage.

In the patient group with residual breast cancer, ypT stage, post-NAC ER status, post-NAC Ki-67 index, LN regression grade, ypN stage, and ipN stage were found to be independent prognostic factors for disease-free survival (all *p* < 0.05; Table 5).

**Table 5:** Multivariate analyses of disease-free survival in the patient group with residual breast disease.

Model	Variable	Category	Multivariate analysis		
			HR	95% CI	*p* value
A	ypT stage	T1 vs. T2–4	3.203	1.957–5.240	< 0.001
	Miller-Payne grade	Grade 4 vs. 1–3	0.835	0.315–2.212	0.717
	RCB class	I vs. II–III	2.770	0.589–13.038	0.197
	Lymphovascular invasion^a^	Absent vs. present	0.993	0.600–1.642	0.978
	Estrogen receptor^a^	Positive vs. negative	3.388	1.917–5.989	< 0.001
	Ki-67 index^a^	< 20% vs. ≥ 20%	2.202	1.278–3.796	0.004
	LN regression grade				0.003
	Grade 0		Reference		
	Grade 1		7.280	2.246–23.600	0.001
	Grade 2		5.893	1.956–17.751	0.002
	Grade 3		7.101	2.540–19.851	< 0.001
B	ypT stage	T1 vs. T2–4	2.405	1.447–3.999	0.001
	Miller-Payne grade	Grade 4 vs. 1–3	0.934	0.350–2.491	0.891
	RCB class	I vs. II–III	1.711	0.369–7.946	0.493
	Lymphovascular invasion^a^	Absent vs. present	0.865	0.517–1.448	0.581
	Estrogen receptor^a^	Positive vs. negative	3.993	2.205–7.230	< 0.001
	Ki-67 index^a^	< 20% vs. ≥ 20%	2.015	1.170–3.470	0.012
	ypN stage				< 0.001
	ypN0		Reference		
	ypN1		2.036	1.060–3.911	0.033
	ypN2		2.818	1.406–5.649	0.003
	ypN3		5.831	2.687–12.653	< 0.001
C	ypT stage	T1 vs. T2–4	2.693	1.600–4.533	< 0.001
	Miller-Payne grade	Grade 4 vs. 1–3	1.176	0.441–3.141	0.746
	RCB class	I vs. II–III	2.040	0.479–8.694	0.335
	Lymphovascular invasion^a^	Absent vs. present	0.809	0.490–1.334	0.406
	Estrogen receptor^a^	Positive vs. negative	3.881	2.208–6.825	< 0.001
	Ki-67 index^a^	< 20% vs. ≥ 20%	2.297	1.355–3.892	0.002
	ipN stage				< 0.001
	ipN0		Reference		
	ipN1		5.259	1.810–15.277	0.002
	ipN2		6.781	2.369–19.413	< 0.001
	ipN3		14.853	4.917–44.863	< 0.001

*HR* hazard ratio, *CI* confidence interval, *RCB* residual cancer burden, *ipN stage* initial pathologic N stage.

^a^post-NAC status.

### Prognostic value of number of LNs with complete regression

**Figure 4:**
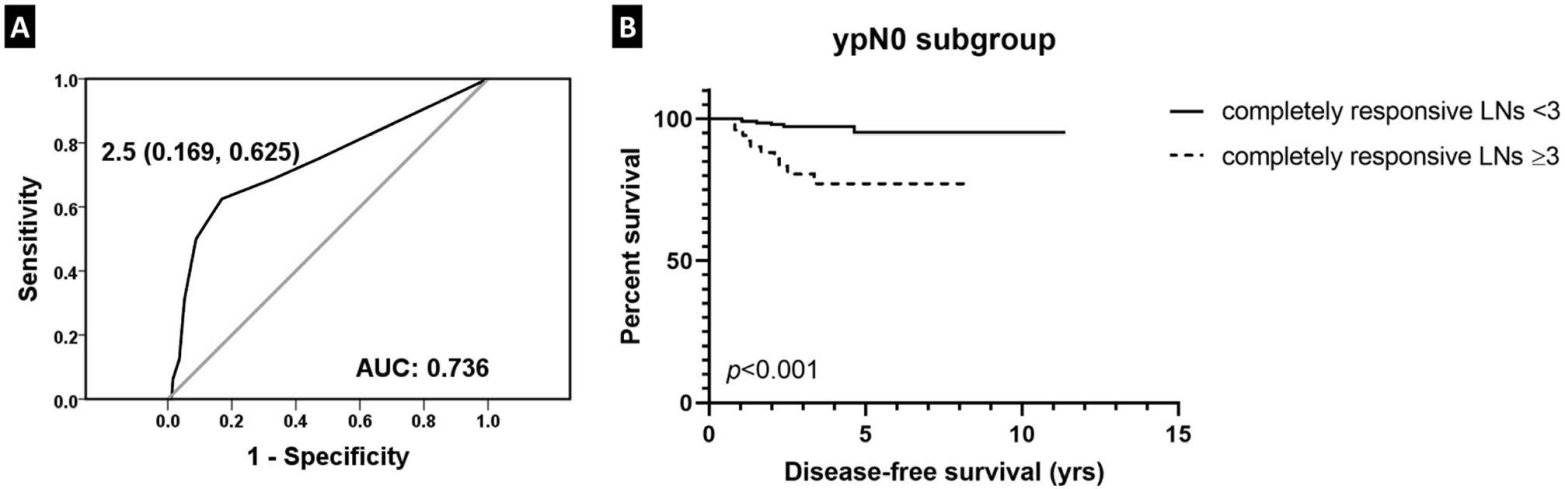
Disease-free survival based on the number of completely-responsive lymph nodes (LNs). **(A)** A receiver operating characteristic curve analysis yields the highest area under curve (AUC) at a value of 2.5 as the cutoff for the number of completely-responsive LNs. **(B)** In ypN0 subgroup, patients with a number of completely-responsive LNs less than 3 show longer disease-free survival compared to those having a completely-responsive LN number greater than or equal to 3.

In patients with no metastatic LNs after NAC (ypN0), those belonging to LN regression grade 0 had a longer disease-free survival compared to those in LN regression grade 1 (*p* = 0.049). We performed a receiver operating characteristic (ROC) curve analysis to find the cutoff value for the number of LNs with complete response that maximized the sum of sensitivity and specificity in predicting tumor recurrence (AUC = 0.736; Fig. 4). Using a cutoff value of 2.5, comparison of patients with a number of completely-responsive LNs less than 3 with those having a completely-responsive LN number greater than or equal to 3 revealed a more favorable clinical outcome of the former compared to the latter (*p* < 0.001; Fig. 4).

### Prognostic value of ratio of non-responsive LNs

**Figure 5:**
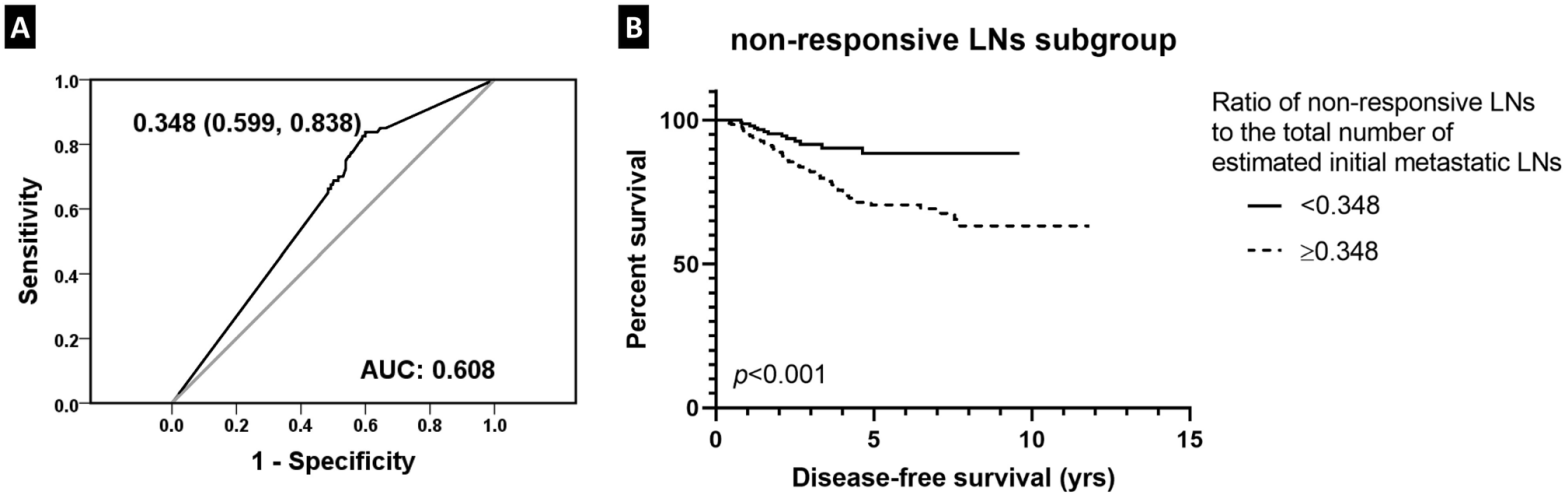
Disease-free survival based on the ratio of non-responsive lymph nodes (LNs) to the estimated total metastatic LNs. **(A)** A receiver operating characteristic curve analysis yields the highest area under curve (AUC) at a value of 0.348 as the cutoff for the ratio of non-responsive LNs to the estimated total initial metastatic LNs. **(B)** Patients with a ratio of non-responsive LNs to the total initial metastatic LNs less than 0.348 show a significantly longer disease-free survival compared to those with a ratio over 0.348.

We also performed a ROC curve analysis to find the cutoff value of the ratio of non-responsive LNs to the total number of estimated metastatic LNs before treatment that maximized the sum of sensitivity and specificity in predicting tumor recurrence in patients with presumed metastatic LNs prior to NAC (AUC = 0.608; Fig. 5). As a result, 0.348 was identified as the cutoff value. Survival analysis revealed that patients with a ratio of non-responsive LNs to the total number of metastatic nodes less than 0.348 had a significantly longer disease-free survival compared to those with a ratio over 0.348 (*p* < 0.001; Fig. 5).

## Discussion

Prognosis of breast cancer patients receiving NAC is associated with two factors at large: tumor biology and tumor response to chemotherapy in the breast and/or axilla. In this study, we focused on the second factor, that is, nodal response to NAC as a potential prognostic factor. Researchers have evaluated the axillary status using several different parameters: absolute number of metastatic LNs after NAC (reflected in pathologic N stage, ypN), size of metastasis, LN ratio (ratio of metastatic LNs among total number of excised LNs), clinical N stage, and extracapsular extension^9,12–15^. However, among the factors related to LN status post-NAC, the prognostic value of LN regression pattern has not been studied widely in breast cancer. In as early as 2003, Newman et al.^16^ reported that detection of treatment effect in axillary LNs could identify patients with an outcome intermediate between node-positive and node-negative patients. However, this study had included only 71 cases to deduce statistically-significant conclusions. Also, in their survival analysis, all patients who showed nodal regression were grouped into the same group regardless of presence or absence of metastasis. In this study, we observed that patient survival was associated with LN regression grade with the best outcome belonging to grade 0 and the worst outcome belonging to grade 3.

It has been reported that NAC downstages the axillary nodal status, and the rate of negative conversion ranges as widely as 20 to 60%^17–19^. Studies have shown that patients who undergo a negative conversion of the axillary nodes have excellent survival regardless of residual breast disease^7,18,20–22^. On a similar note, Newman et al. reported that patient survival was dependent on not only node positivity vs. negativity but also on whether the nodes were downstaged^16^. In the present study, 129 (22.9%) patients underwent a negative nodal conversion (ypN0 with regression, LN regression grade 1). Fayanju et al. reported that patients with a node-positive disease at presentation who achieve a pCR have a prognosis comparable to those who are clinically node-negative at presentation^23^. However, we observed a significant difference in survival between LN regression grade 0 (node-negative at presentation) and LN regression grade 1 (negative nodal conversion) with grade 0 having a longer disease-free survival as a whole. Such discrepancy in results might be due to the difference in study population since Fayanju’s study included only those with clinical N0 and N1 patients and excluded those who showed a discordant or partial response in the breast and axilla. Furthermore, in further analyses, we found that prognostic significance of negative nodal conversion differed according to ER status. While LN regression grade 1 revealed poorer disease-free survival compared to LN regression grade 0 in ER-positive subset, LN regression grades 0 and 1 showed similar favorable prognosis in ER-negative subset. Furthermore, ER-negative breast cancer showed significant difference in survival between regression grade 1 and 2, whereas ER-positive tumors revealed no survival difference between them. Although the reason for this discrepant result by ER status remains unclear, it may be partly explained by the fact that in previous studies, pCR has been consistently reported to be associated with improved survival in ER-negative breast cancers including HER2-positive and triple-negative subtypes, but not in luminal A subtype^24,25^. Further studies are warranted to see if the prognostic significance of LN regression pattern does depend on ER status or breast cancer subtype.

In the ypN0 group, we compared patient survival according to the number of completely-responsive LNs and observed that patients with a completely-responsive LN number less than 3 had a more favorable outcome than those with a completely-responsive LN number greater than or equal to 3. It would be reasonable to infer that those with a smaller number of completely-responsive LNs had a smaller number of LN metastasis before NAC, which in turn, would mean less systemic tumor burden that led to improved survival.

In those patients with initial nodal involvement assessed by chemotherapeutic effect in the LNs, we investigated whether the ratio of non-responsive LNs to the total number of estimated metastatic LNs would have a prognostic value. After obtaining the cutoff ratio of 0.348 from ROC curve analysis, we observed that those patients with a non-responsive nodal ratio over 0.348 had poorer outcome compared to those less than 0.348. Such result suggests that in addition to evaluating LN regression pattern after NAC, evaluating the non-responsive LN ratio may provide additional prognostic information in patients who receive NAC.

Our multivariate analysis revealed the following post-NAC factors to be independently prognostic in the NAC setting: ypT stage, RCB class, LN regression grade, and ipN stage. In those with residual cancer in the breast, ypT stage, post-NAC ER status, post-NAC Ki-67 index, LN regression grade, ypN stage, and ipN stage were independent prognostic factors. From the above factors, ypT stage, pN stage, RCB class, Ki-67 index, and ER status have already been reported to be prognostic of patient outcome in breast cancer after NAC^12,15,26,27^. A novel finding worth noting is the prognostic significance of LN regression grade determined by presence or absence of complete LN regression and residual nodal metastasis, and ipN stage determined by adding the number of metastatic LNs and those with a complete regressive change; we believe this study to be the first one to have evaluated such parameters. Our results may support the idea that axillary status represents systemic micrometastatic burden. Initial pathologic N stage may be a more accurate assessment of a person’s systemic micrometastatic burden rather than ypN stage. Noting chemotherapeutic effects in the post-operative LNs and assessing ipN stage may help stratify patient outcome more accurately. Currently, RCB is the most widely-used index that takes into account both the primary tumor bed response and lymph node status. While it may not be feasible to incorporate the categorical lymph node regression grade into the RCB index, adding numerical parameters such as the number of completely-responsive lymph nodes or the ratio of non-responsive lymph node over total initial metastatic lymph nodes to the RCB index may be useful in making a new prognostic index after further investigations.

On a final note, while axillary nodal dissection has the advantage of accurate assessment of lymph node status in breast cancer patients, it can result in unwanted morbidities such as numbness and debilitating lymphedema; this is the rationale for sentinel node biopsy and the more recent targeted axillary dissection where only the pre-marked lymph nodes suspected of having metastasis are excised in addition to the sentinel nodes. It would be interesting to evaluate the prognostic value of the lymph node regression grade in such less extensive lymph node surgery in further studies.

This study has some limitations. First, we evaluated nodal treatment response using a representative hematoxylin & eosin-stained section rather than serial sections in non-sentinel lymph nodes; thus, we may have missed hidden metastatic foci which could have been present in deeper sections. Therefore, ipN stage estimated by adding the number of LNs with complete chemotherapeutic effect and metastatic nodes may not be exactly the same as the true ipN stage. Secondly, while we included only intranodal fibrosis and foamy histiocyte infiltration as representative of treatment effect, other subtle changes such as a thickened LN capsule can also be a treatment response^28^. Third, the follow-up period was relatively short with a median of 40 months to assess overall survival. Lastly, NAC regimen was not homogeneous, and trastuzumab treatment was given as part of NAC regimen in only some HER2-positive breast cancer patients due to reimbursement issues in Korea in the past.

In summary, we investigated the prognostic significance of axillary LN response in breast cancer patients who received NAC. LN regression grade, presumed ipN stage, number of completely-responsive LNs, and ratio of non-responsive LNs stratified clinical outcome of the patients. In multivariate analysis, LN regression grade and presumed ipN stage were revealed as independent prognostic factors. Further large, prospective studies are warranted to confirm whether LN regression and presumed ipN stage should be routinely evaluated and reported in breast cancer patients who receive NAC.

## Methods

### Patients and samples

We used clinicopathological data of 571 consecutive patients with primary breast cancer who underwent breast-conserving surgery or mastectomy after NAC at Seoul National University Bundang Hospital from October 2004 to December 2015, that had been collected in our previous study^29^. After excluding 8 patients who received sentinel node biopsy prior to NAC, 563 patients were finally included in this study. All patients were diagnosed with invasive carcinoma of the breast via core needle biopsy.

Medical records were reviewed to acquire clinical information of the patients including age, sex, initial clinical T and N stage, chemotherapeutic regimen, cycle of NAC, tumor recurrence, and patient survival. Pathologic information was collected from the pathology reports and the following information was recorded: histologic subtype, histologic grade, pathologic T and N stage after NAC, lymphovascular invasion, and pre- and post-NAC biomarker status.

Pathologic response to NAC was evaluated with the Miller-Payne regression grading^4^ and Residual Cancer Burden class^3^. Pathologic complete response (pCR) was defined as the complete disappearance of all invasive tumor cells from the breast and regional LNs regardless of the presence of residual ductal carcinoma in situ in the breast^30^.

This study was approved by the Institutional Review Board of Seoul National University Bundang Hospital (Protocol # B-1601/332-304), and it was conducted in accordance with the Declaration of Helsinki. Informed consent was waived by the same IRB who approved this study.

### Pathologic evaluation of LNs and definition of response

Of the 563 patients, 488 patients (86.7%) received axillary LN dissection with or without sentinel node biopsy, and the remaining 75 patients (13.3%) received sentinel node biopsy only after NAC. The median number of harvested LNs was 20 (range, 1–65). The sentinel nodes were cut in 2 mm intervals and examined in serial sections. LNs other than sentinel nodes were examined in a single representative section.

For this study, LN status after NAC was carefully evaluated. Histologic signs of tumor regression in LNs were defined as intranodal fibrosis and/or foamy histiocyte infiltration obliterating the nodal architecture, similar to previous studies^16,28,31^. On the other hand, the presence of hemosiderin-laden macrophages only was not regarded as a therapeutic effect since a prior procedure such as large core biopsy of breast or fine needle aspiration of LNs can result in similar histology. We counted the number of LNs with complete tumor regression, and using this data, we estimated the initial number of metastatic LNs before NAC. In addition, the ratio of LNs with residual disease to the total number of estimated metastatic LNs before NAC was calculated.

Regression of LNs was graded as follows: grade 0, no metastasis without regression; grade 1, no metastasis with complete regression (complete response); grade 2, metastasis, but complete regression in some LNs (partial response); grade 3, metastasis and no complete regression in LNs (no response). Initial pathologic N stage was estimated by the sum of the number of metastatic LNs and LNs with a complete regressive change.

### Biomarker status and determination of breast cancer subtype

Immunohistochemical results for standard biomarkers including ER, PR, HER2, p53, and Ki-67 had been collected during the previous study^29^. ER and PR were regarded as positive if there were at least 1% positive tumor nuclei^32^. HER2 expression was scored according to 2013 ASCO/CAP guidelines^33^. HER2 status was determined by HER2 in situ hybridization (ISH) for cases that were equivocal on HER2 immunohistochemistry, and HER2 equivocal cases in ISH were regarded as HER2-non-amplified for statistical analysis. For Ki-67 proliferation index, tumors with 20% or more positive cells were regarded as having high indices^34^.

Standard biomarker status was used to determine the breast cancer subtype of the tumors according to the 2011 St. Gallen Expert Consensus as follows: luminal A (ER + and/or PR + , HER2-, Ki-67 < 14%), luminal B (ER + and/or PR + , HER2-, Ki-67 ≥ 14%; ER + and/or PR + , HER2 +), HER2 + (ER-, PR-, HER2 +), and triple-negative subtype (ER-, PR-, HER2-)^35^.

### Statistical analysis

Pearson’s chi-square test or Fisher’s exact test was used to compare clinicopathological features of tumors according to chemo-responsiveness of LNs and corrections for multiple testing were made by Bonferroni method. A receiver operating characteristic (ROC) curve analysis was performed to identify cutoff values for the number of LNs with a complete pathologic response and ratio of LNs with residual disease to estimated total metastatic LNs before treatment that maximized the sum of sensitivity and specificity in predicting tumor recurrence. Disease-free survivals were estimated by Kaplan–Meier survival curves and statistical significances were determined with the log-rank test. Pearson correlation tests were used to assess the associations between LN regression grade, pathologic N stage after NAC, and estimated initial pathologic N stage. Multivariate analysis was performed by Cox proportional hazards regression model with backward stepwise selection method, using covariates that were associated with survival of the patient in the univariate analyses. Hazard ratios (HR) and their 95% confidence intervals (CI) were calculated for each variable. *P* values less than 0.05 were considered statistically significant, and all reported *p* values were two-sided. All statistical analyses were performed using Statistical Package, SPSS version 25.0 for Windows (IBM Corp., ARMONK, NY).

## Data Availability

The datasets used and/or analyzed during the current study are available from the corresponding author on reasonable request.
